# First Touch course—the impact of a nation-wide boot camp on the transition to Surgical residency

**DOI:** 10.1007/s13304-025-02265-3

**Published:** 2025-06-30

**Authors:** Sofia Gaspar Reis, Mário Rui Gonçalves, Constança Azevedo, Ana Ruivo, Gonçalo Guidi, Ricardo Marinho, Joana Pinto Teles, Ana Sofia Domingos, André Luís Borges, Filipe Rodrigues Quintas, Liliana Grilo Miranda, António Oliveira, José Novo de Matos

**Affiliations:** 1 Unidade Local de Saúde do Arco Ribeirinho - Hospital do Barreiro, Barreiro, Portugal; 2 Faculty of Health Sciences, Academic Clinical Center of Beiras, University of Beira Interior, Av. Infante D. Henrique, Covilhã, Portugal; 3 Unidade Local de Saúde de Braga - Hospital de Braga, Braga, Portugal; 4 Surgery Department, Unidade Local de Saúde de Coimbra - Hospital Universitário de Coimbra, Coimbra, Portugal; 5 Faculty of Medicine, University of Coimbra, Coimbra, Portugal; 6 Unidade Local de Saúde de Trás os Montes e Alto Douro - Hospital de Vila Real, E.P.E., Av. da Noruega, Vila Real, Portugal; 7 Unidade Local de Saúde da Região de Aveiro, Aveiro, Portugal; 8 Unidade Local de Saúde Lezíria - Hospital Distrital de Santarém, Santarém, Portugal; 9 Unidade Local de Saúde de Loures-Odivelas - Hospital Beatriz Ângelongelo, Loures, Portugal; 10 Unidade Local de Saúde de Santa Maria, Lisbon, Portugal; 11 Instituto Ciências da Saúde da Universidade Católica Portuguesa, Porto, Portugal; 12 Hospital Privado da Trofa, Vila Real, Portugal; 13 Unidade Local de Saúde São José - Hospital de São José, Lisbon, Portugal

**Keywords:** Resident, Workshops, Hands-on, Training, Surgical education, Soft skills

## Abstract

Surgical residents face numerous challenges and undergo significant changes at the beginning of their residency. Although there are several courses throughout residency, it is difficult to address all of them in a single activity. Boot camps (BC) have been a way for easing this transition period. Our team developed the “First Touch Course” boot camp in 2017 to join all residents of the same year, before they initiate Surgical residency. Since then, 8 editions have been organised (one per year) with the participation of a total of 481 residents. Eighty-four residents have enrolled in the last two editions, mainly from General Surgery, Gynecology and Urology. The present study analysed the 2023 and 2024 editions to evaluate the impact of this BC on residents’ preparedness and confidence to face residency. More than 71% of the participants rated the course as excellent and 100% considered it exceeded their expectations. Almost 100% considered it will have a significant impact and more than 75% felt better prepared to start residency after the course. More than 73% believe that having a laparoscopic simulator will be useful for continuous skills acquisition. This is the first nationwide, multi-speciality boot camp that promotes continuing education, providing each resident with a personal laparoscopy simulator and laparoscopic instruments, allowing for home-based training. This study highlights the importance and impact of this kind of boot camps in preparing residents for the early stages of surgical residency and the impact it may have on basic skills acquisition.

**Graphical Abstract:**

**Figure:**
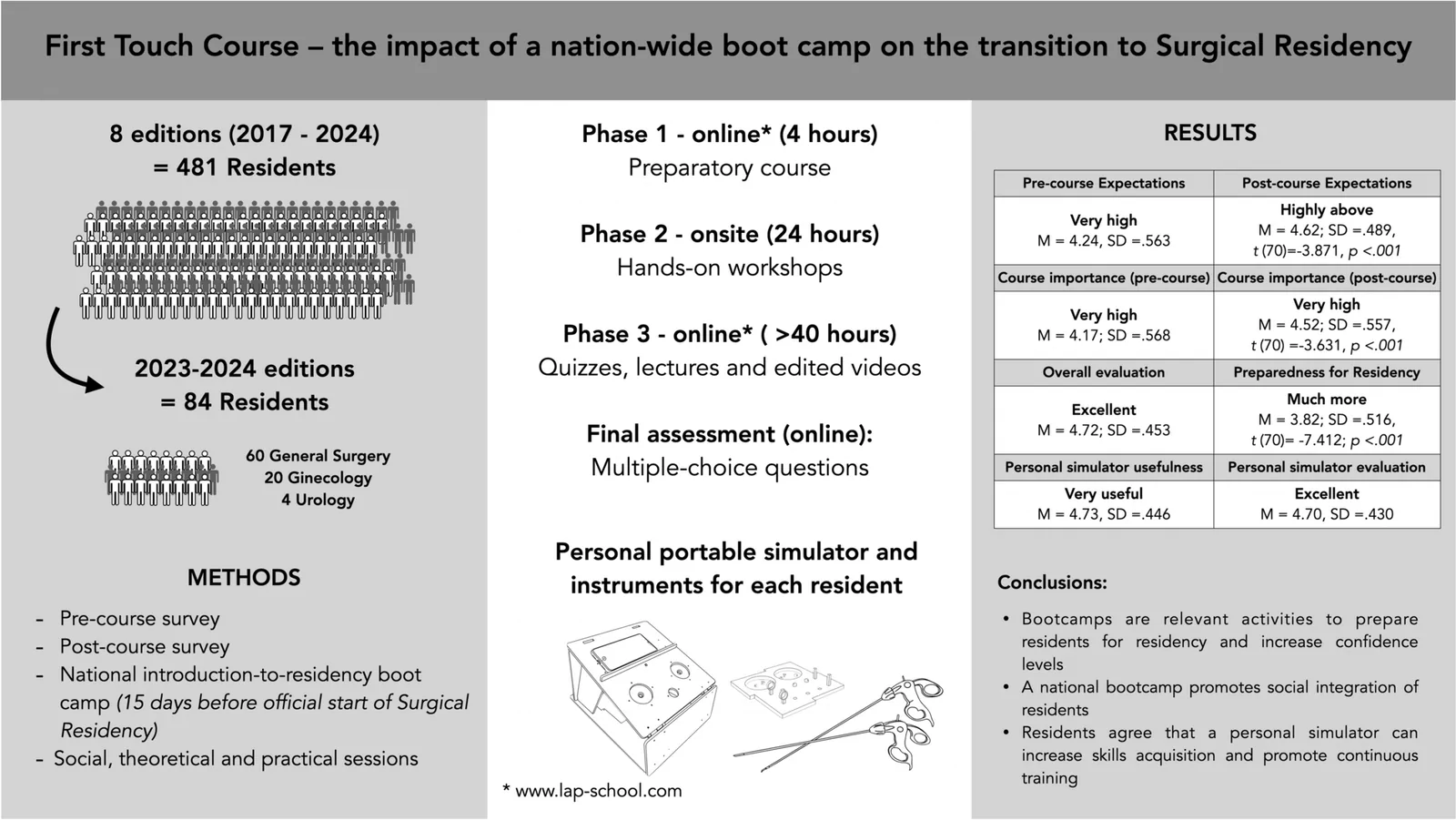

**Supplementary Information:**

The online version contains supplementary material available at https://doi.org/10.1007/s13304-025-02265-3.

## Introduction

**Fig. 1:**
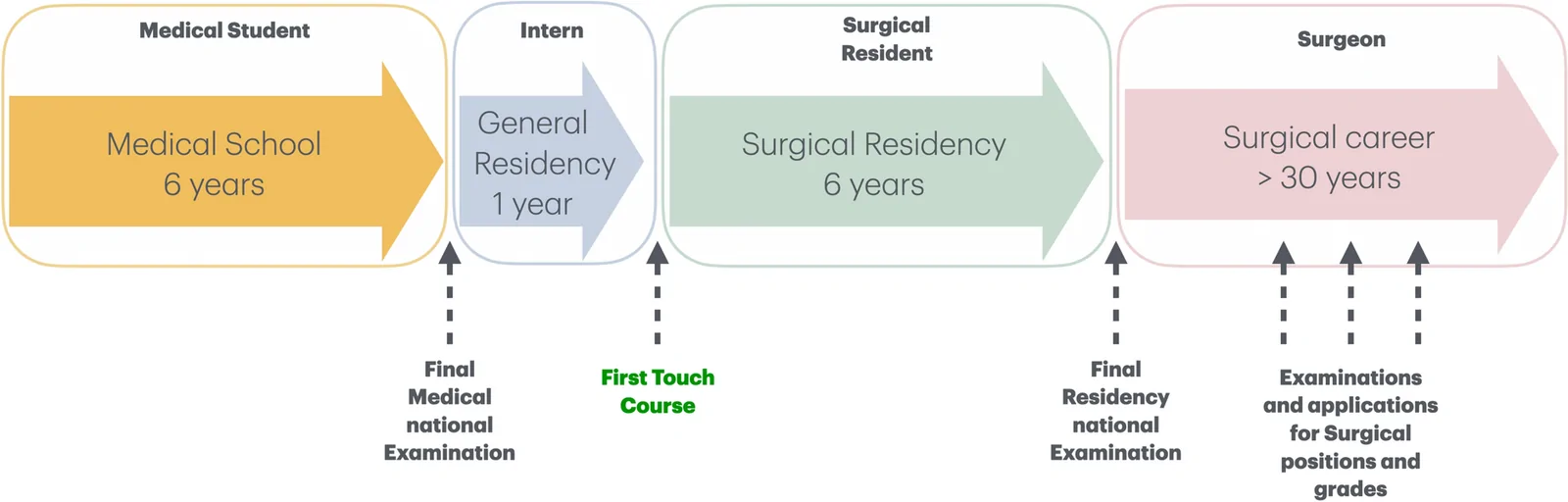
Educational Pathway in Portugal—from Medical Student to Surgeon

Surgical residency profoundly changes all aspects of a doctor’s life. The transition between General and Surgical residency is, in fact, very abrupt and stressful. In Portugal, before Surgical residency, all residents go through 12 months of General residency (Fig. 1).

During General residency they have contact with surgical specialities for a period that lasts from 2 to 6 months. For many of them, this transition means relocation to a different city and hospital with unfamiliar internal protocols and workflows. Besides that, residents also face growing responsibilities, a higher demand for knowledge, skills, and decision-making. Usually, they experience an overwhelming feeling of insecurity, stress, and unpreparedness [1, 2]. Surgical training opportunities are not equal among countries and access to training tools such as simulators and instruments is not widely available. Box trainers and virtual reality simulators help to minimize these difficulties as they can improve access to surgical training and thus to achieve proficiency, especially in laparoscopic and robotic surgery [3–6]. There is a growing emphasis on simulation-based assessment and training to achieve a predetermined skill level and competence, which can motivate doctors to engage in further practice [7, 8]. Although there are several courses for surgical residents, it is difficult to address all of these challenges that residents face at the beginning of residency in a single activity. This led us to develop the “First Touch Course” (FT) boot camp, in 2017.

### The concept of the FT

The FT is a nationwide and annual introduction course to surgical residency. It is all-inclusive, including an onsite and online scientific program, housing, meals, social program, a portable laparoscopy simulator, a set of basic and intermediate exercises plus one Maryland and one clinch grasper. We hypothesized that this format, besides empowering the surgical residents with knowledge and technical skills, would foster social relationship among the participants of the same year. FT covers mainly 3 specialities: General Surgery, Gynecology, and Urology. It is also open to Pediatric, Cardiac and Thoracic Surgery Residents.

The main goals of FT are:

- 1. To welcome all residents to the surgical community, providing opportunities to network with their colleagues, Senior residents and Surgeons (Faculty);
- 2. To offer hands-on, simulation-based training for the most common initial surgical procedures;
- 3. To provide a portable laparoscopy simulator and instruments for home-based continuous practice;
- 4. To be an introduction to Surgical residency boot camp (BC), with lectures and videos covering numerous topics including surgical treatment of surgical pathologies, residency structure, labor rights, and legislation;

We developed a pilot edition of the BC in 2017, only for General Surgery residents. The curriculum covered theoretical lectures and practical skills on surgical topics, running through 3,5 days of lectures and workshops. Additional hours were allocated for team-building activities (4 h) and during meals and at the end of the day some cultural/social activities have been developed (6 h). The original curriculum was repeated in 2018. The positive feedback from participants in both 2017 and 2018 editions generated interest from Gynecology and Urology residents. For the 3rd edition (2019), and continuing thereafter, we expanded registrations to include Gynecology/Obstetrics and Urology as they share similar skills, interests and duties in residency. A specific program has been designed for each group and included in the main program.

In December 2020, it wasn’t possible to organize the onsite session of the course due to the COVID-19 pandemic. Because of the limitations imposed, the project had to face major changes concerning the format. Consequently, our team developed an online school (www.lap-school.com) in November 2020, and the theoretical component was moved online to avoid personal gatherings. Additionally, the curriculum underwent structural changes so that we could deliver it in less days and fully dedicated to hands-on skills training. The new format has been repeated in December 2021 and December 2022. In 2023, gathering all the experience from the six previous editions, we redesigned all the scientific programs, workshops, and the overall structure of the BC to develop our definitive curriculum, which is described and analyzed in the present paper. This format has been repeated in 2024. Over 8 editions, a total of 481 participants from various Portuguese hospitals attended the BC, including 312 in General Surgery, 110 in Gynecology, 42 in Urology, 15 in Pediatric Surgery, and 2 in Thoracic Surgery. According to the concept and aims of the course, we decided that the best date for the BC would be 2 weeks prior to the start of residency (Portuguese Surgical residency starts on January 1st every year). The video-lectures in lap-school.com have an average duration of 15 to 20 min and are grouped in “modules”. These are focused on various topics, that we believed to be the most important ones for new residents. Since the first edition, the course has been advertised by social media (e.g. Facebook, Instagram) and also by word of mouth. The 2024 fee was established at 349€ per participant. The aims of this study are to assess the quality and educational value of this introduction-to-residency BC for new surgical residents; and understand its impact on residents; preparedness and self-perceived confidence levels at the early stages of residency. The authors are Surgeons and Senior Surgical residents that coordinate the whole BC and also participate in Session 2, as leaders of the workshops.

## Methods

### Online surveys

Two online surveys have been developed using Google forms® platform, and sent before (pre-course survey) and just immediately after the last workshop (post-course survey). The pre-course survey aimed to assess demographics; previous contact with surgical specialities; residents’ confidence and preparedness for residency; and the main expectations about the course. The post-course survey aimed to analyze the global evaluation and utility of the course; the workshops; the laparoscopic simulator; and to assess residents’ perception of the boot camp’s impact on confidence and preparedness for residency. Both questionnaires included open, closed and multiple-choice questions. Data received was analyzed anonymously.

### The FT curriculum

The curriculum is divided into three sessions: online preparatory course, FT boot camp and online theoretical course. Session 1 and 3 (online) are accessible through all the 6-year period of residency, so Residents can view and review lectures, videos, etc. as many times as they need.

#### Session 1: online preparatory course

This session is carried out on our online school (www.lap-school.com) and it covers theoretical content that is essential for the in-person workshops, with a duration of almost 2 h. Completing this session is mandatory prior to session 2 (onsite) (Table 1).

**Table 1:** Online lectures of the course (Session 1 and Session 3)

Online Module 1	General surgery	Urology	Gynecology
(This Pre-course module corresponds to Session 1) Average duration—15 min video lectures	Theme 1: Minor Surgical Procedures - Asepsis - Local Anesthesia - Threads and needles - Knots and suture - Chest Tube Placement – Indications and technique - Central Venous catheter placement – Indications and technique		

Online Module 2			
(These lectures correspond to Session 3) Average duration—15 min video lectures	Theme 1: Emergencies in Surgery I - Acute cholecystitis - Acute appendicitis - Acute pancreatitis - Acute diverticulitis - Digestive hemorrhage - Intestinal occlusion - Perforation of hollow viscera - Perianal pathology	Theme 1: Introduction to Urology I - Mandatory/optional internships and internships abroad: what should I do? - Laparoscopic surgery in Urology	Theme 1 – Gynecological Emergencies - Abnormal Uterine Bleeding - Pelvic Pain - Vulvovaginitis - Pelvic Inflammatory Disease
	Theme 2: Emergencies in Surgery II - 5 clinical cases	Theme 2: Introduction to Urology II - Approach to the urological patient - Urological emergencies - Introduction to emergency surgery in Urology - Basic procedures in Urology - Outpatient surgery in Urology: basic surgical techniques - Introduction to Endourology	Theme 2 – Obstetric Emergencies - Hemorrhage in the 1st trimester - Hemorrhage in the 2nd and 3rd trimester - Hypertensive Disorders of Pregnancy - Pregnancy Intrahepatic Cholestasis - Obstetric Emergencies
	Theme 3: Emergencies in Surgery III - Hemorrhoidal Thrombosis Incision – pathology, indications and video - Abscess incision and drainage—pathology, indications and video		Theme 3 – Breast Pathology - ABC of Benign Breast Pathology - ABC of Malignant Breast Pathology
	Theme 4: Emergencies in Surgery IV - The polytrauma patient – ​​types of shock - Trauma – what to think about on the way to the emergency room? – Initial approach to ABCDE - Damage control trauma surgery		Theme 4 – Infertility - Introduction to medically assisted reproduction techniques
			Theme 5 – Pelvic Anatomy - Pelvic Anatomy
			Theme 6 – Complementary Exams in Gynecology - Gynecological Ultrasound

Online Module 3			
Introduction to the Surgical Patient (These lectures correspond to Session 3) Average duration—15 min video lectures	Theme 1: Introduction to the Surgical Patient—Pre-operative I - Pre-anesthetic evaluation—The anesthesiologist's point of view - Surgical risk stratification - Concept, advantages and inclusion criteria in outpatient surgery - Hypocoagulation and reversal - Risk of venous thromboembolism and prophylaxis		Theme 1: Introduction to the Surgical Patient—Pre-operative I - Pre-anesthetic evaluation - Pre operative appointment - Risk of venous thromboembolism and prophylaxis
	Theme 2: Introduction to the Surgical Patient—Pre-operative II - Operative preparation - The Upper GI surgeon's point of view - The Bariatric surgeon's point of view - The Colorectal surgeon's point of view - The HBP surgeon's point of view - The Abdominal wall surgeon's point of view - The Thyroid surgeon's point of view - The Outpatient surgeon's point of view - The Robotic surgeon's point of view		
	Theme 3: Introduction to the Surgical Patient- Pre-operative III - Introduction to the ERAS program in General Surgery - Assessment of nutritional status – global approach		

Module 4			
Introduction to the Surgical Patient - Intraoperative (These lectures correspond to Session 3) Average duration—15 min video lectures	Theme 1: Our first surgeries step by step - Laparoscopic cholecystectomy - Laparoscopic appendectomy - Anterior inguinal hernioplasty - Posterior inguinal hernioplasty - Excision of Pilonidal cyst Theme 2: Introduction to the operating room I - Incisions in General Surgery - Drains in General Surgery - Ostomies and precautions to be taken in their construction		Theme 1 – Gynecological Surgery - Abdominal and Laparoscopic Hysterectomy - Vaginal Hysterectomy - Stress Incontinence Surgery - Surgical Therapy for Pelvic Organ Prolapse Correction OASIS, Lacerations and their correction
	Theme 3: Introduction to the operating room II - Surgical Table - Asepsis in the operating room		

Module 5			
Introduction to the Surgical Patient - Post-operative (These lectures correspond to Session 3) Average duration—15 min video lectures	Theme 1: Post-operative - Postoperative complications – The importance of the operative report! - Therapeutic table – How to minimize complications?		Theme 1: Post-operative - The operative report! and therapeutic table - Pain Control
	Theme 2: Complications		Theme 2: Complications
	- Non-surgical complications of surgical patients! - Complications in the immediate postoperative period - Early surgical complications - Late surgical complications - Systematic recording of complications – Clavien-Dindo classification		- Complications vascular, intestinal, urinary and nervous - Surgical infections and prophylactic antibiotic therapy

Module 6			
Introduction to the practical session (These lectures correspond to Session 3) Average duration—15 min video lectures	Theme 1: Minimally invasive surgery- Laparoscopy – Positioning of the patient and the team - Pneumoperitoneum - Electrosurgery		Theme 1: Minimally invasive surgery - Ergonomics and the operating Room - Surgical Exposure - Electrosurgery

#### Session 2: The “FT Boot camp”—2.5-day onsite course

**Fig. 2:**
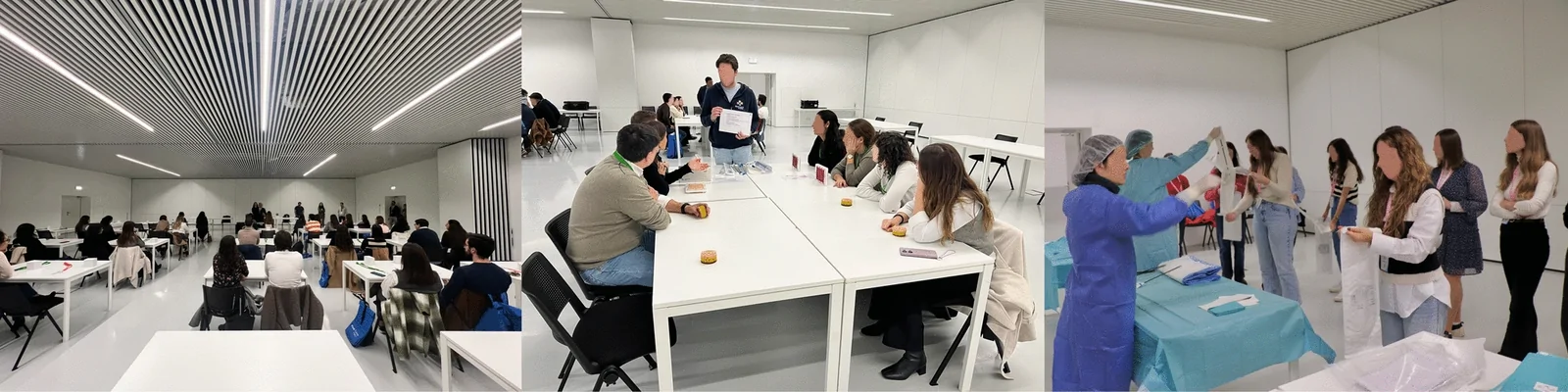
General and specific workshops

This session is entirely dedicated to hands-on practice. Each workshop is supervised by a dedicated instructor, either a senior Resident (> PGY 6) or a Surgeon, ensuring that participants receive adequate guidance and support throughout the sessions (Fig. 2).

**Fig. 3:**
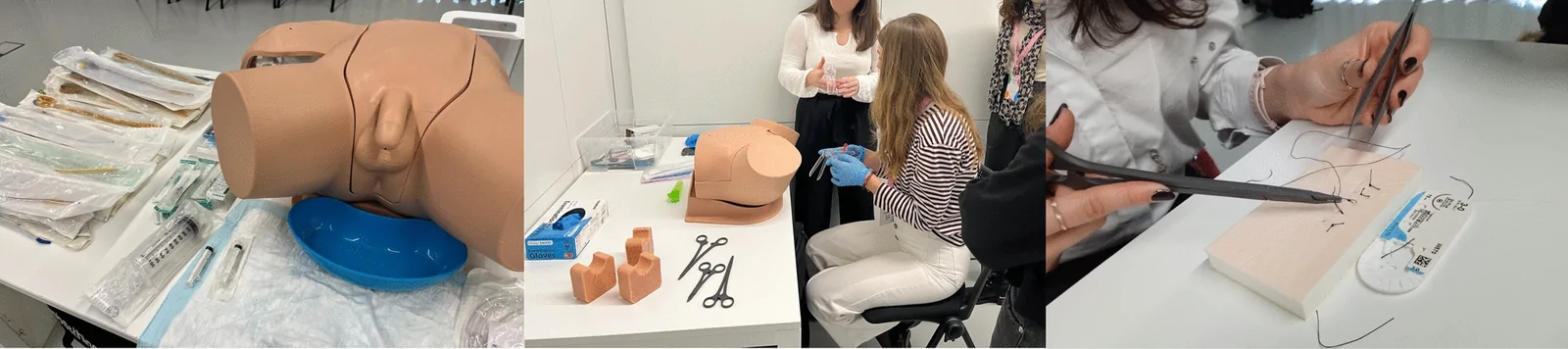
Examples of the models used during the workshops

These workshops last from 1 to 2 h and formative and summative feedback is provided. A review of indications and contraindications for each procedure, relevant anatomy, procedural kit and equipment management, sterilization technique, and simulation are included (Fig. 3). The stations and workshops with the models used and the learning objectives are detailed in Table 2. Usually the activities start at 08:30 and there are 2 coffee breaks and 1 lunch, per day.

**Table 2:** Workshops, models and learning objectives

	Specialty	Model and material	Learning objectives	Duration	Faculty	Ratio trainer/trainees	Evaluation
Suture training workshop	All	- Suture pad (Limbs and things^®^) - Surgical instruments (multi-brand) - Sutures (Ethicon^®^)	Presentation of surgical material used in minor surgery situations; - How to approach the wounds; - Types of threads and different sutures, their indication and how to perform them; - Suture training	1 h	Senior Residents (> PGY 6) and Surgeons	Between 1:4 and 1:10 ratio	Formative and Summative feedback for all sessions
Operating room workshop	All	- Mannequin - OR surgical drapes (^®^Molnlycke) - Surgical instruments (multi-brand)	Exposure to materials in the surgical center (surgical gowns, gloves, surgical material, surgical fields…); - Explanation and training on putting on surgical clothing; - Training of placement of surgical tables and wards	1 h	Senior Residents (> PGY 6) Surgeons and Nurses	Between 1:4 and 1:10 ratio	
Laparoscopy Training	All	- First trainer simulator (FTacademy^®^) - Laparoscopic instruments (FTacademy^®^ ) - Basic and Intermediate exercises pad (FTacademy^®^)	Exposure to laparoscopic material; - Training of manual dexterity, motor skills and coordination in laparoscopy; - Laparoscopic suture training	3 h	Surgeons	Between 1:4 and 1:10 ratio	
CVC Training Workshop	- General Surgery - Urology	- CVC model (FTacademy^®^) - CentralLineMan^®^ and FemoralLineMan^®^Models (Medical Simulator^®^) - Commercial catheters (Bbraun^®^) - Ultrasound (Fujifilm^®^)	Central catheterization material display; - Understanding indications and contraindications of central catheterization; - Understanding the anatomy of the cervical, subclavian and femoral regions; - Training in the placement of central catheters using ultrasound guidance and anatomical references	1 h	Senior Residents (> PGY 6) Surgeons	Between 1:4 and 1:10 ratio	
Chest tube training Workshop	- General Surgery - Urology	- Chest tube model (FTacademy^®^ Simulator^®^) - Commercial chest tubes (Bbraun^®^) - Surgical instruments (Bbraun^®^)	Chest tube material display; - Understanding indications and contraindications of chest tubes; - Understanding the anatomy of the thoracic region; - Training in the placement of chest tubes using anatomical references	1 h	Senior Residents (> PGY 6) Surgeons	Between 1:4 and 1:10 ratio	Formative and Summative feedback for all sessions
Airway management Workshop	- General Surgery - Urology	- Orotracheal intubation models - Orotracheal tubes (Bbraun^®^)	Exhibition of material used in airway protection (Guedel tubes, laryngeal mask, endotracheal tubes…) - Airway approach in trauma situations; - Orotracheal intubation training on mannequins	1 h	Anethesists	Between 1:4 and 1:10 ratio	
Introduction to Nutrition Workshop	- General Surgery - Urology	- Commercial nutrition products (Baxter^®^)	Learning about the different types of nutrition (enteral, parenteral); - Understanding the usefulness of parenteral and enteral nutrition, as well as the calculations required for their application; - Care in prescribing nutrition to ensure the patient's basic needs	1 h	Surgeons	1:16 ratio	
Female and male catheterization training Workshop	- Urology	- Model (Limbs and things^®^) - Foley catheter (Bbraun^®^)	Knowledge of female and male urogenital anatomy; - Display of the different materials to be used when performing a genital catheterization; - Female and male catheterization training	2 h	Senior Residents (> PGY 6) and Surgeons	1:2 ratio	
Scrotal examination Workshop	- Urology	- Model for scrotal palpation (Medical Simulator^®^)	Knowledge of female and male urogenital anatomy; - Introduction to scrotal pathology; - Palpation of healthy anatomical models and those with malignant scrotal pathology	2 h	Senior Residents (> PGY 6) and Surgeons	Between 1:4 and 1:10 ratio	
Gynecological examination workshop	- Ginecology	- Model for uterine palpation (Medical Simulator^®^)	Knowledge of female uterine anatomy; - Introduction to uterine pathology; - Palpation of healthy anatomical models and those with malignant uterine pathology	1 h	Senior Residents (> PGY 6) and Surgeons	Between 1:3 to 1:5 ratio	Formative and Summative feedback for all sessions
Obstetric examination and ultrasound	- Ginecology	- Human volunteer (Pregnant woman) - Ultrasound (Fujifilm^®^)	Introduction to obstetric ultrasound with the aim of enabling: - Confirm gestational age; - Check for adequate fetal growth; - Check the position of the placenta; - Determine the craniocaudal length, biparietal diameter, head circumference and femur length - Know how to look for and identify fetal developmental abnormalities; - Ultrasound training in pregnant volunteers	1 h	Senior Residents (> PGY 6) and Surgeons	Between 1:3 to 1:5 ratio	
Laceration management	- Ginecology	- Laceration model (FTacademy^®^) - Surgical instruments (multi-brand)	Understanding of female pelvic anatomy and associated injuries during childbirth; - Learning about episiotomies and pelvic lacerations; - Pelvic laceration repair training on anatomical models	1 h	Senior Residents (> PGY 6) and Surgeons	Between 1:3 to 1:5 ratio	
IUD training	- Ginecology	- Model (Bayer^®^) - Speculum (Bayer^®^) - IUD (Bayer^®^)	Display of the material needed to place the intrauterine device (IUD); - Indications and contraindications for the different types of IUD; - IUD placement training	1 h	Senior Residents (> PGY 6) and Surgeon	Between 1:3 to 1:5 ratio	
IMPLANON Training	- Ginecology	- Model (Organon^®^) - Implanon (Organon^®^)	Display of the material needed to place the Implanon; - Indications and contraindications for the Implanon; - Implanon placement training and certification	1 h	Senior Residents (> PGY 6) Surgeons	Between 1:3 to 1:5 ratio	Formative and Summative feedback for all sessions

**Fig. 4:**
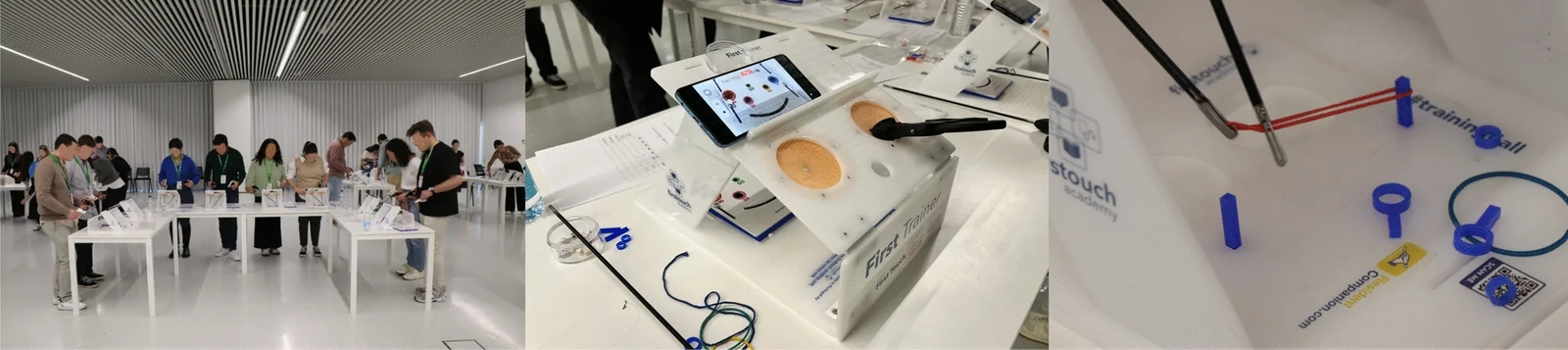
Laparoscopic surgery workshop and the First Trainer simulator

The final workshop of the course is an introduction to laparoscopy workshop. Before the final workshop all the residents receive their First Trainer^®^, an accessible/low-cost, lightweight and portable training box, and 2 surgical instruments—1 maryland and 1 clinch grasper. Residents receive comprehensive instruction on the use of the First Trainer, principles of laparoscopic ergonomics and triangulation, as well as technical aspects of the laparoscopic instruments. Various exercises and tasks are used to this purpose (Fig. 4). Following this workshop, residents engage in a hands-on laparoscopic competition that consists of scoring the maximum number of points in each exercise, on multiple rounds, while the time to perform the task is reduced. This adds pressure in two dimensions: competition and time.

The top performers on the laparoscopy contest are selected to participate in the FT Championship. This last competition challenges residents to complete designated tasks within a specified time frame and allows for the assessment of their performance and the skills learned during the whole BC. The resident who demonstrates the highest level of proficiency and completes the tasks in the shortest time is declared the winner and receives a prize as recognition of his/her achievement. After Session 2, a certificate is issued to every participant.

#### Session 3: theoretical online course

The third and final session of the course has more than 40 hours of theoretical lectures and is carried out on www.lap-school.com. These lectures are available since the moment the resident registers in the course and during the 6 years of residency. The contents of this part can be consulted in Table 1.

There are online multiple-choice question examinations after every Session 3 module (Table 1).

When the participants complete the 3 Sessions, an online certificate is automatically issued by the www.lap-school.com platform.

### Statistical analysis

All anonymous data were analyzed using SPSS (IBM SPSS Statistics for Windows, Version 30.0). Descriptive data were presented as frequencies, means and standard deviation. Paired-samples t-Student tests were performed to compare means between pre and post-intervention surveys.

## Results

A total of 84 residents participated in the BC, 39 in 2023 and 45 in 2024. The group was composed of 60 new General Surgery residents (71,4%), 20 new Gynecology residents (23,8%) and 4 new Urology residents (4,8%).

### Survey response rates and participants demographics

Seventy-eight participants (*n* = 78, response rate 92,9%) answered to the pre-intervention survey, with 66,6% females (*n* = 52) and 33,4% males (*n* = 26). The mean age of the population was 26,28 years (24–39) and 55 participants were future residents of General Surgery (70,5%), 19 of Gynecology residents (24,4%) and 4 of Urology (5,1%) (Table 3).

**Table 3:** Characteristics of the Participants

	Pre-course				Post-course			
	Total n		n	%	Total n		n	%
Gender	78	Male Female	26 52	33.3 66.7	71	Male Female	22 49	31 69
Age (years)		Mean	26.28			Mean	26.14	
		24 25 26 27 29 30 31 33 34 39	3 38 21 7 3 1 1 1 1 2	3.8 48.7 26.9 9.0 3.8 1.3 1.3 1.3 1.3 2.6		24 25 26 27 29 30 31 39	3 35 19 7 3 1 1 2	4.2 49.3 26.8 9.9 4.2 1.4 1.4 2.8
Speciality		General Surgery Urology Ginecology	55 4 19	70.5 5.1 24.4		General Surgery Urology Ginecology	48 4 19	67.6 5.6 26.8
MIS experience		None	78	100		None	71	100
Group		FT 2023 FT 2024	38 40	51,3 48,7		FT 2023 FT 2024	34 37	47.9 52.1

Seventy-one participants (n = 71, response rate of 84,5%) answered to the post-intervention survey, with 69% females (*n* = 49) and 31% males (*n* = 22). The mean age of the population was 26,52 years (24–39) and 48 participants were future residents of General Surgery (67,6%), 19 of Gynecology (26,8%) and 4 of Urology (5,6%) (Table 3).

### Expectations about the boot camp

Before the course, 93.6% (*n* = 73) of the respondents considered that both the expectations regarding their participation in the course and the importance of residency were “High/very high” (*M* = 4.24; SD = 0.563 and *M* = 4.17; SD = 0.568, respectively) (Table 4).

**Table 4:** Global expectations, utility and evaluation

Results	Value	Precourse					Postcourse					Comparison		
		Total N	N	%	M	SD	Total N	N	%	M	SD	t-test	df	Sig
Course expectations	3—Neither high or low 4—High expectations 5—Very High expectations	78	5 49 24	6.4 62.8 30.8	4.24	.563	–	–	–	–	–	− 3.871	70	< .001
	4—Above my expectations 5—Very above my expectations	–	–	–	–	–	71	27 44	50.7 49.3	4.62	.489			
Utility of Workshops	4—High utility 5—Very high utility	–	–	–	–	–	71	17 54	23.9 76.1	4.76	.430	–	–	–
Workshops evaluation	4—Good 5—Excellent	–	–	–	–	–	71	23 48	32.4 67.6	4.68	.471	–	–	–
Course evaluation	4—Good 5—Excellent	–	–	–	–	–	71	20 51	28.2 71.8	4.72	.453	–	–	–
First trainer evaluation	3—Neither good or bad 4—Good 5—Excellent	–	–	–	–	–	71	1 19 51	1.4 26.8 71.8	4.70	.490	–	–	–
First Trainer utility in the future	4—High utility 5—Very High utility	–	–	–	–	–	71	19 52	26.8 73.2	4.73	.446	–	–	–
Importance for Residency	2—Low importance 3—Neither low or high importance 4—High importance 5—Very High importance	78	1 4 54 19	1.3 5.1 69.2 24.4	4.17	.568	71	0 2 30 39	0 2.8 42.3 54.9	4.52	.557	− 3.631	70	< .001
Preparedness for Residency	1—Totally disagree 2—Disagree 3—Neither agree or disagree 4—Agree 5—Totally agree	78	9 19 31 17 2	11.5 24.4 39.7 21.8 2.6	2.79	.998	71	0 0 17 50 4	0 0 23.9 70.4 5.6	3.82	.516	− 7.412	70	< .001

Sixty-two percent (*n* = 44) of the respondents considered that the BC has been “Highly above” their initial expectations (*M* = 4,61; SD = 0.490) and all of them would recommend the course to other residents (100%, *n* = 71).

The three features of the BC considered as the most important aspects were the laparoscopy simulator (80%, *n* = 57); the hands-on workshops (77,5%, *n* = 55); and the social networking with their new colleagues (52,1%, *n* = 37).

### Course evaluation

The full results of the global evaluation of the workshops and the course can be found in Table 4. Concerning the individual evaluation and impact of each workshop, the majority rated them very positively (Table 5).

**Table 5:** - Evaluation and impact of the workshops

		Value	n	%	Mean	SD
Suture training	Evaluation	3—Neither good or bad 4—Good 5—Excellent	9 29 33	12.7 40.8 46.5	4.34	.696
	Impact	3—Neither low or high impact 4—High impact 5—Very High impact	1 20 31	4 35 32	4.39	.597
Operating room	Evaluation	2—Bad 3—Neither good or bad 4—Good 5—Excellent	1 7 37 26	1.4 9.9 52.1 36.6	4.24	.686
	Impact	2—Low impact 3—Neither low or high impact 4—High impact 5—Very High impact	3 5 28 35	4.2 7.0 39.4 49.3	4.34	.792
Laparoscopy Training	Evaluation	3—Neither good or bad 4—Good 5—Excellent	3 14 54	4 20 76	4.72	.539
CVC Training Workshop	Evaluation	3—Neither good or bad 4—Good 5—Excellent	2 16 34	3.8 30.8 65.4	4.62	.565
	Impact	3—Neither low or high impact 4—High impact 5—Very High impact	1 20 31	1.9 38.5 59.6	4.56	.608
Chest tube training Workshop	Evaluation	2—Bad 3—Neither good or bad 4—Good 5—Excellent	1 4 25 22	1.9 7.7 48.1 42.3	4.31	.701
	Impact	2—Bad 3—Neither good or bad 4—Good 5—Excellent	2 3 25 22	3.8 5.8 48.1 42.3	4.29	.750
Airway management Workshop	Evaluation	3—Neither good or bad 4—Good 5—Excellent	8 24 20	15.4 46.2 38.5	4.23	.703
	Impact	2—Low impact 3—Neither low or high impact 4—High impact 5—Very High impact	1 10 34 7	1.9 19.2 65.4 13.5	3.90	.634
Introduction to Nutrition Workshop	Evaluation	2—Bad 3—Neither good or bad 4—Good 5—Excellent	1 11 16 24	1.9 21.2 30.8 46.2	4.21	.848
	Impact	3—Neither low or high impact 4—High impact 5—Very High impact	4 26 22	7.7 50 42.3	4.35	.623
Female and male catheterization training Workshop	Evaluation	4—Good 5—Excellent	1 3	25 75	4.75	.500
	Impact	4—High impact 5—Very High impact	1 3	25 75	4.75	.500
Scrotal examination Workshop	Evaluation	4—Good 5—Excellent	1 3	25 75	4.75	.500
	Impact	4—High impact 5—Very High impact	1 3	25 75	4.75	.500
Gynecological examination Workshop	Evaluation	4—Good 5—Excellent	1 18	5.3 94.7	4.95	.229
	Impact	4—High impact 5—Very High impact	2 17	10.5 89.5	4.89	.315
Obstetric examination and ultrasound	Evaluation	2—Bad 3—Neither good or bad 4—Good 5—Excellent	1 0 3 15	5.3 0 15.8 78.9	4.68	.749
	Impact	3—Neither low or high impact 4—High impact 5—Very High impact	1 4 14	5.3 21.1 73.7	4.68	.582
Laceration management	Evaluation	3—Neither good or bad 4—Good 5—Excellent	4 7 8	21.1 36.8 42.1	4.37	.761
	Impact	4—High impact 5—Very High impact	1 5 13	5.3 26.3 68.4	4.68	.478
IUD training	Evaluation	3—Neither good or bad 4—Good 5—Excellent	3 6 10	15.8 31.6 52.6	5	.000
	Impact	3—Neither low or high impact 4—High impact 5—Very High impact	6 13	31.6 68.4	5	.000
IMPLANON Training	Evaluation	5—Excellent	19	100	4.21	.787
	Impact	5—Very High impact	19	100	4.63	.597

All of the participants (*n* = 71) evaluated the workshops positively (*M* = 4.68; SD = 0.471) and considered that they will be useful for residency (*M* = 4.76; SD = 0.430). Regarding the overall opinion of the laparoscopy simulator, 71.8% (*n* = 51) of the participants rated it as “Excellent” (*M* = 4.70; SD = 0.490) and 73.2% (*n* = 52) considered it will be "Very useful” during residency for skills acquisition (*M* = 4.73; SD = 0.446).

Regarding the overall opinion of the course 71,8% (*n* = 51) rated the course as “Excellent” (*M* = 4.72; SD = 0.453). After the course, the residents’ opinion on the importance of the course has increased and almost 55% (*n* = 39) totally agreed that the BC will be “Very important” for their future residency (*M* = 4.52; SD = 0.557, t (70) = − 3.631; p < 0.001) (Table 4).

### Self-perceived preparedness for residency

The course significantly improved residents’ self-perceived preparedness for residency and more than 75% of the residents felt more or much more prepared by the end of the BC (*M* = 3.82; SD = 0.516, t (70) = − 7.412; *p* < 0.001)—(Table 4).

### Self-perceived confidence

The full results of both the pre and post-course self-perceived confidence can be found in Tables 6, 7 and 8 (also see Supplementary file 1–4).

**Table 6:** Confidence levels—Basic and General Surgery Workshops

Confidence levels	Speciality	Value	Precourse					Postcourse					Comparison		
			Total N	N	%	M	SD	Total N	N	%	M	SD	t-test	df	Sig
Suturing and Wound Workshop	All	1—Very low confidence 2—Low confidence 3—Neither low or high confidence 4—High confidence 5—Very High confidence	78	3 8 23 27 17	3.8 10.3 29.5 34.6 21.8	3.60	1.061	71	0 0 0 36 35	0 0 0 50.7 49.3	4.49	.504	-6.822	70	< .001
Operating room Workshop	All	1—Very low confidence 2—Low confidence 3—Neither low or high confidence 4—High confidence 5—Very High confidence	78	3 5 29 39 12	3.8 6.4 24.4 50.0 15.4	3.67	.949	71	0 1 4 33 33	0 1.4 5.6 46.5 46.5	4.38	.663	-6.035	70	< .001
Laparoscopy Workshop	All	1—Very small Improvement 2—Small improvement 3—Neither small or big improvement 4—Big improvement 5—Very big improvement	-—-	-—-	-—-	-—-	-—-	71	0 1 13 41 16	0 1.4 18.3 57.7 22.5	4.01	.686	-—-	-—-	-—-
CVC training Workshop	General Surgery Urology	1—Very low confidence 2—Low confidence 3—Neither low or high confidence 4—High confidence 5—Very High confidence	59	38 15 3 1 2	64.4 25.4 5.1 1.7 3.4	1.54	.934	52	2 8 15 23 4	3.8 15.4 28.8 44.2 7.7	3.37	.971	-11.290	51	< .001
Chest tube training Workshop	General Surgery Urology	1—Very low confidence 2—Low confidence 3—Neither low or high confidence 4—High confidence 5—Very High confidence	59	45 9 4 1 0	76.3 15.3 6.8 1.7 0	1.34	.685	52	2 6 25 17 2	3.8 11.5 48.1 32.7 3.8	3.21	.848	-13,549	51	< .001
Airway management Workshop	General Surgery Urology	1—Very low confidence 2—Low confidence 3—Neither low or high confidence 4—High confidence 5—Very High confidence	59	42 5 8 1 3	71.2 8.5 13.6 1.7 5.1	1.61	1.114	52	1 4 21 24 2	1.9 7.7 40.4 46.2 3.8	3.42	.776	-13.549	51	< .001
Nutrition Surgical patient Workshop	General Surgery Urology	1—Very low confidence 2—Low confidence 3—Neither low or high confidence 4—High confidence 5—Very High confidence	59	20 26 11 2 0	33.9 44.1 18.6 3.4 0	1.92	.816	52	0 0 19 26 7	0 0 36.5 50 13.5	3.77	.675	-12.194	51	< .001

**Table 7:** Confidence levels—Gynecology

Confidence levels	Value	Precourse					Postcourse					Comparison		
		Total N	N	%	M	SD	Total N	N	%	M	SD	t-test	df	Sig
Gynecological examination Workshop	1—Very low confidence 2—Low confidence 3—Neither low or high confidence 4—High confidence 5—Very High confidence	19	1 6 6 6 0	5.3 31.6 31.6 31.6 0	2.89	.937	19	0 0 1 12 6	0 0 5.3 63.2 31.6	4.26	.562	-5344	18	< .001
Obstetric examination and ultrasound Workshop	1—Very low confidence 2—Low confidence 3—Neither low or high confidence 4—High confidence 5—Very High confidence	19	14 5 0 0 0	73.7 26.3 0 0 0	1.26	.452	19	3 6 8 2 0	15.8 31.6 42.1 10.5 0	2.47	.905	-6.172	18	< .001
Perineal laceration repair Workshop	1—Very low confidence 2—Low confidence 3—Neither low or high confidence 4—High confidence 5—Very High confidence	19	17 2 0 0 0	89.5 10.5 0 0 0	1.11	.315	19	3 5 9 2 0	15.8 26.3 47.4 10.5 0	2.53	.905	-6.444	18	< .001
IUD training Workshop	1—Very low confidence 2—Low confidence 3—Neither low or high confidence 4—High confidence 5—Very High confidence	19	16 2 1 0 0	84.2 10.5 5.3 0 0	1.21	.535	19	1 1 7 10 0	5.3 5.3 36.8 52.6 0	3.37	.831	-11.275	18	< .001
Implanon training Workshop	1—Very low confidence 2—Low confidence 3—Neither low or high confidence 4—High confidence 5—Very High confidence	19	8 4 6 1 0	42.1 21.1 31.6 5.3 0	2.00	1.000	19	0 0 2 9 8	0 0 10.5 47.4 42.1	4.32	.671	-9.551	18	< .001

**Table 8:** Confidence levels—Urology

confidence levels	Value	Precourse					Postcourse					Comparison		
		Total N	N	%	M	SD	Total N	N	%	M	SD	t-test	df	Sig
Urinary Catheterisation training Workshop	Very low confidence Low confidence Neither low or high confidence High confidence Very High confidence	4	2 0 2 0 0	50 0 50 0 0	2.00	1.155	4	0 0 0 2 2	0 0 0 50 50	4.50	.577	− 3.873	3	.015
Scrotal examination Workshop	Very low confidence Low confidence Neither low or high confidence High confidence Very High confidence	4	2 1 1 0 0	50 25 25 0 0	1.75	.957	4	0 0 0 0 4	0 0 0 0 100	5.00	.000	− 6789	3	.003

#### Pre-course

After General residency, 91% (*n* = 71) considered to be confident when performing open suture; 21,8% (*n* = 17) were very confident when performing elective minor surgeries; and 15,4% (*n* = 12) considered to be very confident to participate in the Operating room (Table 6).

Considering both the future General Surgery and Urology residents (*n* = 59), the great majority felt “Low/very low confidence” regarding the placement of a central venous catheter (89,8%), of a chest tube (91,6%), and the performance of an orotracheal intubation (79,7%). Almost 80% of the participants felt unconfident about nutrition in the surgical patient (Table 6).

Regarding the future Gynecology/Obstetrics residents (*n* = 19), 31,6% were confident when performing a gynecological examination. On the other side, all of them (100%) felt unconfident when performing an obstetric evaluation or a repair of a perineal laceration. Most of them were not confident to insert an Intrauterine Device (IUD) or an Implanon® (94,7%, 63,2%, respectively)—(Table 7).

Regarding the specific Urological workshops (*n* = 4), the future Urology residents did not feel confident placing an urinary catheter or performing a scrotal examination alone (100%)—(Table 8).

#### Post-course

When comparing levels of perceived confidence, there was a significant improvement from before to after the course in all workshops, for all specialities. Comparative means and significance can be consulted on Tables 6, 7 and 8 (also see Supplementary file 1–4).

##### Minor surgery and operating room workshops

At the end of these workshops, there was a significant improvement in confidence among the residents (p < 0.001) (Table 6).

##### Laparoscopy workshop

More than 80% (*n* = 57) of the trainees felt a “Big/Very big improvement” on their laparoscopic skills (*M* = 4,01; SD = 0.686) (Table 6).

##### General Surgery specific Workshops

At the end of these workshops, a significant improvement was observed and the majority of the residents felt “Confident/very confident” in regard to the procedures (*p* < 0.001) (Table 6).

##### Ginecology specific workshops

A significant improvement was observed and the majority of the residents felt “Confident/very confident” in regard to the gynecological examination, the IUD placement and the Implanon^®^ placement (*p* < 0.001). Although the improvements on the obstetric examination and perineal laceration workshops were significant (*p* < 0.001), the majority of the residents still felt low confidence with these procedures (Table 7).

##### Urology specific workshops

All respondents considered to be “Confident/Very confident” when performing an urinary catheterization or performing a scrotal examination (*p* = 0.015 and *p* = 0.003, respectively)—(Table 8).

## Discussion

Although it varies among Countries, to become a Surgeon it is mandatory to go through internships and Surgical residency. In Portugal, the transition between General and Surgical residency happens from one day to another, usually on the 1st of January. The challenges faced by these young doctors are numerous and make them experience a feeling of insecurity, stress, and unpreparedness [9–11] on a crucial phase that lays the foundation for their future careers and professional development in surgery [12]. A new position as a surgical resident means facing challenges in 6 main domains of life: personal; professional; responsibility and accountability; scientific knowledge (cognitive); technical skills (psychomotor); and social (affective). The course surveys provided valuable insights into participants' perceptions and experiences from the First Touch course. Previous to the course, residents pointed out that “Receive a portable laparoscopic simulator” was the most important feature of the course, followed by “To be prepared for residency” and “Contact with my new colleagues”. These aspects may correspond to the level 4 to 6 (cognitive, psychomotor and affective) in which the FT aims to have an important influence.

### Cognitive level

When designing the curriculum we selected workshops that allow training and simulation of usual and frequent procedures, characteristic of the early stages of residency. Furthermore, these workshops are specifically selected and developed for each speciality. Our study showed that this fact increased residents’ engagement, highlighted by the excellent evaluation of the BC. We believe it has also influenced the self-confidence improvement related to these procedures. This significant improvement on confidence resulted on an improved sense of global preparedness to face the real-world challenges of surgical residency.

### Psychomotor level

Concerning the 5th domain (psychomotor), the ability to train and learn from one's mistakes in a controlled and secure environment underscores the essential importance of surgical simulation as the most effective approach for enhancing psychomotor skills and achieving technical proficiency—all without exposing patients to any risk [3, 13, 14]. Training and simulation yield significant improvements across a range of medical and surgical procedures [15]. Box trainers, simulators and simulation models have played a major role in surgical education but there are yet some limitations and challenges [4, 16]. One of the strengths of the FT is the laparoscopy hands-on session with the First Trainer and the possibility for the residents to take it home so they can continue to practice [5]. The overall opinion on our simulator was very positive. The great majority of the residents considered that having the laparoscopic simulator and instruments will be very usefull for their future, which corroborates our hypothesis.

### Affective level

Lastly, the 6th domain in which the transition to residency has an important impact is the social aspect of Residents’ life (affective domain). In recent years, BC have shown that can be a valuable solution for residents integration. They gather residents together, fostering contact and network and explore simulation-based activities to improve skills and knowledge acquisition [17–19]. In Portugal, a national BC for anesthesiology residents—“InAnestesia”—that gathers all the residents of the same year for an introduction to residency, is running since 2011 [20]. In 2014, the University of Toronto established a BC focused on Thoracic Surgery, which received positive feedback from participants, directors, and faculty [21]. In 2016, Rábago JL, et al. published the outcomes of a simulation-based introductory course to anesthesia and found that it has been increased self-reported efficacy and led to transfer skills from simulation to clinical practice [22]. Also in 2016, Cleland et al. implemented the first surgical bootcamp in the UK. It ran over 4 days and included training in non-technical, communication and operative surgical skills. Like in our case, formal social events were incorporated into the program and informal socialization among learners was encouraged. The authors found that a surgical BC can deal with social and cultural processes as well as the usual individual, cognitive and acquisitive learning [23]. In 2023, Buscail et al. developed an immersive operating room BC aiming to create awareness about anesthesiology and surgical specialities among sixth-year of medical students. Students found the BC very useful and considered that it worked as a way of changing preconceived notions about these specialities [24]. Sadati et al. in 2024, developed a surgical bootcamp for first-year surgical residents in General surgery, orthopedics, neurosurgery, and gynecology. Like in our case, they implemented a multi-speciality BC although we only focused on specialities that perform laparoscopy and work on the abdominal cavity. The authors found that their surgical BC played a crucial role in enhancing the satisfaction, knowledge, and competence of surgical residents [25]. 

Despite the fact that many BC have been described, it is difficult to address all of these challenges that residents face at the beginning of residency on a single activity [10]. An all-inclusive BC such as the FT promotes even further the contact between the residents and can improve the feeling of belonging to the new community. Additionally, in every edition of our BC we create a specific WhatsApp® group and we invite all the attendants to sign in. From our experience, this greatly increases networking and promotes a lasting effect during residency.

## Strenghts

**Fig. 5:**
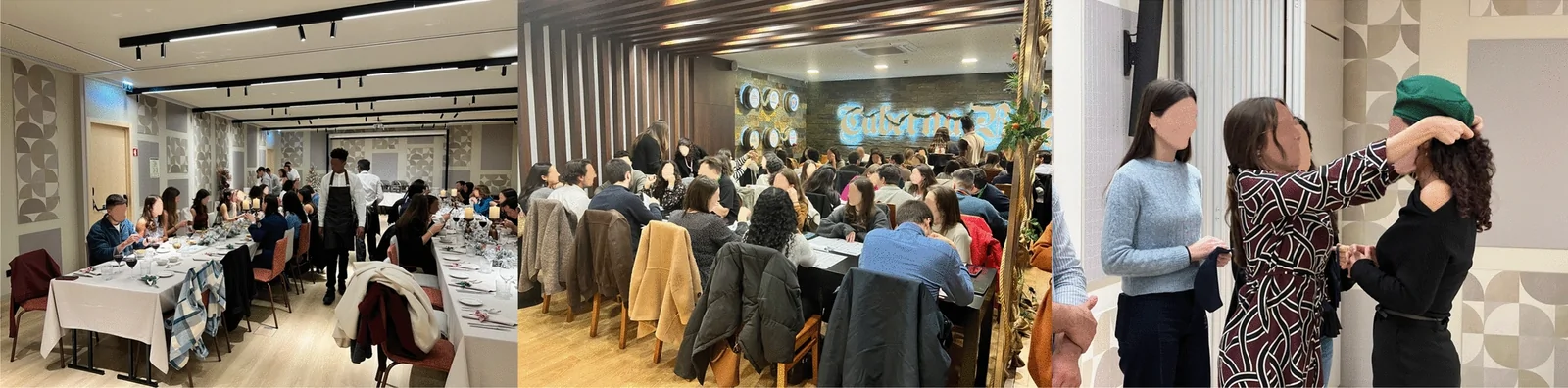
Social activities during the course

We believe that our Boot camp is really innovating in some aspects that make it important for other research or surgical teams to implement future boot camps. In fact, our BC is the first and the only one that offers a personal simulator and laparoscopic instruments to each participant to take home and keep practicing to improve minimally invasive surgery skills. We believe this is game changing in regard to increase access to MIS training. Additionally, we also think that other aspects of our BC are very important, positive and different from the ones previously published:

- 1 - This is a nationwide boot camp
- 2 - It is targeted at new surgical residents
- 3 - As an introduction to residency BC, it happens just 15 days before the beginning of residency allowing residents to be more prepared for the “first day”; to know other colleagues of the same year; and meet senior residents and surgeons (our Faculty) thus entering this national community established since 2017. These interactions are favoured by various social and team-building activities during the course—Fig. 5.

Finally, we consider that another differentiating aspect of our BC in comparison to others is that it is a part of a much more complete learning activity, that includes 3 sessions, two of them online and the most important one, onsite, fully hands-on. These 3 sessions complement each other and increase residents’ engagement and the outcomes of the course.

## Conclusions

Transition from General to Surgical residency is a stressful period for Residents. To diminish the impact on the cognitive, psychomotor and affective domains during this period, it is important to stimulate and support the participation of Residents in training activities since the first moment. As far as we know, this is the first, all-inclusive, introduction to residency course that aims to bring together all the new residents just before the start of their residency. This concept increases opportunities for networking, enhances skill acquisition and promotes continuous practice “at home” by including a portable laparoscopy simulator to every participant [26]. The analysis of the last 2 editions of the BC confirmed that it has had a positive impact over the last 8 years on how residents face the beginning of residency, enhancing their sense of preparedness and increasing their confidence in important procedures. The social impact of a BC like the FT is enormous and it has a lasting effect on residents during all the residency period, promoting network and a sense of belonging within the surgical community. This study brings new data to support that these national boot camps should be mandatory and that residents should have protected time and financial support to attend them. These promising results open up exciting prospects for future research in this area and the expansion of such BC to other medical and surgical specialities, even outside of Portugal. Our next steps and future research will focus on evaluating the long-term impact of this BC throughout the six years of residency.

## Supplementary Information

Below is the link to the electronic supplementary material.

## Data Availability

All data access is granted upon request to the authors.

## References

[1] Bamford R, Langdon L, Rodd CA, Eastaugh-Waring S, Coulston JE (2018) Core trainee boot camp, a method for improving technical and non-technical skills of novice surgical trainees. A before and after study. Int J Surg 57:60–65 29653248 10.1016/j.ijsu.2018.03.083

[2] Perez AR, Boscardin CK, Pardo M (2022) Residents’ challenges in transitioning to residency and recommended strategies for improvement. J Educ Perioper Med 24:E679 35707017 10.46374/volxxiv_issue1_boscardin PMC9176399

[3] Seymour NE, Gallagher AG, Roman SA, O’Brien MK, Bansal VK, Andersen DK, Satava RM (2002) Virtual reality training improves operating room performance: results of a randomized, double-blinded study. Ann Surg 236:458–463 12368674 10.1097/00000658-200210000-00008 PMC1422600

[4] Gravante G, Venditti D (2013) A systematic review on low-cost box models to achieve basic and advanced laparoscopic skills during modern surgical training. Surg Laparosc Endosc Percutan Tech 23:109–120 23579503 10.1097/SLE.0b013e3182827c29

[5] Thinggaard E, Konge L, Bjerrum F, Strandbygaard J, Gögenur I, Spanager L (2017) Take-home training in a simulation-based laparoscopy course. Surg Endosc 31:1738–1745 27515838 10.1007/s00464-016-5166-5

[6] Seltzer H, Swayze E, Thottathil L, Dewey J, Jabara J, Mehta A, Frederick J, Yousif P, Parikh S, Tsuei A, Miller L, Linares LS (2023) The impact of homemade laparoscopic box trainers on medical student surgical skills: a randomized control pilot study. Surg Innov 30:84–93 35499271 10.1177/15533506221094956

[7] Gonçalves MR, Morales-Conde S, Gaspar Reis S, Carlos Alves P, Novo de Matos J, Oliveira A, Marinho R, Cadime I, Castelo-Branco Sousa M (2024) RAWS4all project: validation of a new silicone model for robotic TAPP inguinal hernia repair. Surg Endosc 38:1329–1341 38110794 10.1007/s00464-023-10592-y PMC10881695

[8] Gonçalves M, Mück B, Faure J-P, Topart P, Castelo-Branco M (2024) RoboticSurgery4all: are discovery courses important for robotic surgery skills acquisition? J Robotic Surg 18:322 10.1007/s11701-024-02077-4 PMC11324769 39141249

[9] Abuhusain H, Chotirmall S, Hamid N, O’Neill S (2009) Prepared for internship? Ir Med J 102:82–84 19489196

[10] Mattar SG, Alseidi AA, Jones DB, Jeyarajah DR, Swanstrom LL, Aye RW, Wexner SD, Martinez JM, Ross SB, Awad MM, Franklin ME, Arregui ME, Schirmer BD, Minter RM (2013) General surgery residency inadequately prepares trainees for fellowship: results of a survey of fellowship program directors. Ann Surg 258(440):9 10.1097/SLA.0b013e3182a191ca 24022436

[11] Chang LY, Eliasz KL, Cacciatore DT, Winkel AF (2020) The transition from medical student to resident: a qualitative study of new residents’ perspectives. Acad Med 95:1421–1427 32349016 10.1097/ACM.0000000000003474

[12] Hannon FB (2000) A national medical education needs’ assessment of interns and the development of an intern education and training programme. Med Educ 34:275–284 10733724 10.1046/j.1365-2923.2000.00512.x

[13] Aggarwal R, Darzi A (2011) Simulation to enhance patient safety: why aren’t we there yet? Chest 140:854–858 21972381 10.1378/chest.11-0728

[14] Gonçalves MR, Novo de Matos J, Oliveira A, Marinho R, Cadime I, Carlos Alves P, Morales-Conde S, Castelo-Branco M (2023) Robotic4all project: results of a hands-on robotic surgery training program. Laparoscopic, Endoscopic Robotic Surg 6:1–8

[15] Lawaetz J, Soenens G, Eiberg J, Van Herzeele I, Konge L, Nesbitt C, Gentile F, Stavroulakis K, Weiss S, Nayahangan LJ (2023) Facilitators and barriers to implementation of simulation based education in vascular surgery in Europe. Eur J Vasc Endovasc Surg 66:428–436 37330202 10.1016/j.ejvs.2023.06.009

[16] Vitish-Sharma P, Knowles J, Patel B (2011) Acquisition of fundamental laparoscopic skills: is a box really as good as a virtual reality trainer? Int J Surg 9:659–661 21964217 10.1016/j.ijsu.2011.08.009

[17] Minter RM, Amos KD, Bentz ML, Blair PG, Brandt C, D’Cunha J, Davis E, Delman KA, Deutsch ES, Divino C, Kingsley D, Klingensmith M, Meterissian S, Sachdeva AK, Terhune K, Termuhlen PM, Mullan PB (2015) Transition to surgical residency: a multi-institutional study of perceived intern preparedness and the effect of a formal residency preparatory course in the fourth year of medical school. Acad Med 90:1116–1124 25785673 10.1097/ACM.0000000000000680

[18] Green CA, Huang E, Zhao NW, O’Sullivan PS, Kim E, Chern H (2018) Technical skill improvement with surgical preparatory courses: what advantages are reflected in residency? Am J Surg 216:155–159 29117916 10.1016/j.amjsurg.2017.10.037

[19] Kassam AF, Singer KE, Winer LK, Browne D, Sussman JJ, Goodman MD, Makley AT (2021) Acquisition and retention of surgical skills taught during intern surgical boot camp. Am J Surg 221:987–992 32981654 10.1016/j.amjsurg.2020.09.018

[20] Gomes SH FT, Guimarães R, Oliveira V, Arantes S, Sousa N, Pêgo JM (2012) InAnestesia – Introduction to Clinical Anesthesiology—2 years in review. 18th Annual Meeting of Society for Simulation in Europe (SESAM), Stavanger, Norway

[21] Schieman C, Ujiie H, Donahoe L, Hanna W, Malthaner R, Turner S, Czarnecka K, Yasufuku K (2018) Developing a national, simulation-based, surgical skills bootcamp in general thoracic surgery. J Surg Educ 75:1106–1112 29246590 10.1016/j.jsurg.2017.11.008

[22] Rábago JL, López-Doueil M, Sancho R, Hernández-Pinto P, Neira N, Capa E, Larraz E, Redondo-Figuero CG, Maestre JM (2017) Learning outcomes evaluation of a simulation-based introductory course to anaesthesia. Rev Esp Anestesiol Reanim 64:431–440 28347552 10.1016/j.redar.2016.12.008

[23] Cleland J, Walker KG, Gale M, Nicol LG (2016) Simulation-based education: understanding the socio-cultural complexity of a surgical training “boot camp.” Med Educ 50:829–841 27402043 10.1111/medu.13064

[24] Buscail E, Bolzinger M, Shourick J, Lier ML, Sautier P, Delhoste M, Muscari F, Carrère N, Maulat C, Dalmas Y, Brinas P, Prudhomme T, Podio C, Houze-Cerfon CH, Rauzy O, Geeraerts T, Abbo O (2024) Welcome to the operating theatre! Introductory bootcamp in operating theatre specialities for medical students. BMC Med Educ 24:1263 39501215 10.1186/s12909-024-06165-9 PMC11539667

[25] Sadati L, Karami S, Edalattalab F, Hajati N, Azarsina S, Nouri Khaneghah Z, Abjar R (2024) Designing, implementing, and evaluating a basic surgical skills bootcamp: an effective approach to enhance competency in surgical residency training. The Surgeon 22:e208–e212 39174363 10.1016/j.surge.2024.08.008

[26] Gonçalves MR, Marinho R, Reis SG, Viveiros R, Teixeira MM, Andrade AK, de Carmo GM, Rodrigues PP, Castelo-Branco SM (2025) LapAppendectomy4all: validation of a new methodology for laparoscopic appendectomy simulation and training. Updates Surg. 10.1007/s13304-025-02127-y 39922944 PMC12263465

