# Supplementary material for: First Touch course—the impact of a nation-wide boot camp on the transition to Surgical residency
Source: Updates Surg. 2025 Jun 30;78(5):2291–311. doi: 10.1007/s13304-025-02265-3 (PMC13538175; doi:10.1007/s13304-025-02265-3)
Supplement: Supplementary file 1 [file 13304_2025_2265_MOESM1_ESM.docx]

**F**

**E**

**D**

**C**

**B**

**A**

Supplementary file 1 - Pre and Post-confidence - Global and General Surgery Workshops.

A) Minor Surgery and Suture; B) Operating room; C) Central venous catheter; D) Chest Tube; E) Airway management; F) Nutrition
