# Supplementary material for: First Touch course—the impact of a nation-wide boot camp on the transition to Surgical residency
Source: Updates Surg. 2025 Jun 30;78(5):2291–311. doi: 10.1007/s13304-025-02265-3 (PMC13538175; doi:10.1007/s13304-025-02265-3)
Supplement: Supplementary file 2 [file 13304_2025_2265_MOESM2_ESM.docx]

**D**

**A**

**B**

**C**

**E**

Supplementary file 2 - Pre and Post-confidence - Gynecology Workshops.

A) Gynecological examination; B) Obstetric examination; C) Perineal laceration repair; D) IUD placement; E) Implant placement
