# Supplementary material for: First Touch course—the impact of a nation-wide boot camp on the transition to Surgical residency
Source: Updates Surg. 2025 Jun 30;78(5):2291–311. doi: 10.1007/s13304-025-02265-3 (PMC13538175; doi:10.1007/s13304-025-02265-3)
Supplement: Supplementary file 3 [file 13304_2025_2265_MOESM3_ESM.docx]

**A**

**B**

Supplementary file 3 - Pre and Post-confidence - Urology Workshops

A) Urethral catheterization; B) Scrotal examination
